# Impacts of Built-Environment on Carbon Dioxide Emissions from Traffic: A Systematic Literature Review

**DOI:** 10.3390/ijerph192416898

**Published:** 2022-12-16

**Authors:** Ying Huang, Yongli Zhang, Feifan Deng, Daiqing Zhao, Rong Wu

**Affiliations:** 1 School of Engineering Science, University of Science and Technology of China, Hefei 230026, China; 2 Guangzhou Institute of Energy Conversion, Chinese Academy of Sciences, Guangzhou 510640, China; 3 School of Architecture and Urban Planning, Guangdong University of Technology, Guangzhou 510062, China

**Keywords:** built environment, traffic carbon emissions, influencing factors, knowledge map

## Abstract

With the acceleration of global urbanization, the interaction between the urban built environment and transportation carbon emissions (TCE) has become an urgent problem and an area of intensive research. This paper presents a bibliometric and visual analysis of 1060 pieces of literature related to the built environment and TCE from 1998 to 2022. It explores the current research progress and future development trends in this field. The results show the following. (1) The number of papers published on the built environment and TCE during this period has shown a continuous increasing trend, and the periods of growth can be divided into three stages. (2) Research in this area has been carried out in many countries and regions around the world, involving different dimensions such as examinations at the city, provincial, and national levels. (3) Through an analysis involving keyword clustering, a keyword hotspot map, and a burst map, we have established that the research on TCE has exhibited step-by-step growth, and the carbon emissions from vehicles is the topic that has been considered over the longest period. (4) The impact of the built environment on TCE can be broadly divided into macro-functional and micromorphological factors.

## 1. Introduction

In recent years, severe changes in the global climate and frequent extreme weather events and natural disasters have primarily been attributed to human energy consumption and carbon emissions [1,2,3]. If greenhouse gas (GHG) emissions are not reduced, these trends of environmental change will affect global ecosystems and human society and threaten sustainable development [4,5]. As places that provide various services, facilities, and living accommodation, cities are the main areas in which carbon emissions are generated. With the acceleration of urbanization, an increasing number of people continue to gather in urban areas. By 2050, the world population may be close to 9 billion, and 68% of these people will live in urban areas [6]. The activities of urban residents lead to notable carbon emissions, of which transportation, as an indispensable part of urban residents’ daily lives, is inevitably one of the primary sources. In particular, private car travel not only results in traffic congestion [7] and energy problems in increasing numbers of cities [8,9,10], but also poses great challenges to public health (e.g., increased incidence rate, road traffic injuries and air pollutions) [11,12,13]. Globally, the transport sector accounts for 15% of GHG emissions [14] and 24% of carbon dioxide (CO_2_) emissions from energy consumption [15]. In addition, CO_2_ accounts for 76% of total GHG emissions [14], and it is predicted that global transport-related CO_2_ emissions will increase by 50% by 2030 and more than 80% by 2050 [16]. Passenger vehicles (e.g., cars and buses) account for about 45.1% of total CO_2_ emissions [15]. Hence, research into the impact of carbon emissions from urban traffic is significant for developing a global low-carbon economy [17].

The daily activities of urban residents are conducted in the built environment, and it thus has a long-term impact on TCE [18,19,20]; it has therefore attracted the attention of many researchers worldwide [21,22,23,24]. Many studies have shown that changes in the built environment are closely related to changes in urban TCE [25,26], and this is a complex process with both direct and indirect effects. From a theoretical point of view, most researchers believe that the relationship between the built environment and TCE is regulated by changes in individual travel behavior and motor-vehicle ownership as intermediary factors. Therefore, a large number of studies have measured the relationship between the built environment and personal travel behavior and motor-vehicle ownership [27,28,29,30]. With the gradual improvement of the “3D” elements of the built environment (diversity, density, design) into “6D” elements (diversity, density, design, destination accessibility, traffic distance, and demand management), there are many pieces of literature that discuss factors such as density, land use, and accessibility.

On the whole, although there have been many studies examining the impact of the built environment on urban TCE, the following problems still need to be further ad-dressed. (1) A systematic analysis framework needs to be established because there are currently many different research indicators in use. There may be differences in the results obtained using different approaches and measures. (2) Due to the randomness and complexity of residents’ personal travel behavior, most existing reports discuss the influence of the built environment in relation to physical space. Hence, it is not easy to accurately quantify different individuals’ diverse travel needs.

In this work, we divided the built environment’s impact on TCE into two perspectives: the macro influences of functional features and the micro effects of morphological features. The former mainly involves the impact of the land-use structure and land-use mix on regional functions; the latter explicitly discusses the characteristics of road length and road network density, and the quantities and distribution of traffic and service facilities. We also summarize the impact of differences in analysis methods relating to the built environment in different countries around the world. In this way, through a systematic review, we hope to provide a scientific reference for future research on the impact of the built environment on TCE.

## 2. Materials and Methods

### 2.1. Data

In this work, we took the core collection of Web of Science (WOS) as reference to search the global literature on the built environment and TCE from 1998 to 2022 (data as of August 2022). The topics of this paper are “#1:TS=land use, built environment, and urban form” and “#2:TS=TCE, transportation carbon emissions, travel carbon emissions, and transport carbon emissions.” A total of 2280 search results were obtained. After secondary screening and deduplication, 1060 articles with high relevance to the topic were obtained and output in text format.

### 2.2. Methods

Various visualization tools can be used to display the content of the research field in the form of graphics and provide researchers with a means for analysis using multivariable and dynamic visualizations [31,32]. Among these, CiteSpace and VOSviewer are widely used. To date, these tools have been applied to visual analysis in a variety of disciplines, including road safety [33], chemistry [34], climate-change studies [35], education [36], medical big data [37], and philosophy [38]. In this work, the powerful literature-analysis functions of VOSviewer and CiteSpace were used as the main analysis tools. In addition, based on bibliometric analysis, many cited pieces of literature were read to evaluate and summarize the main research contents and trends related to the current worldwide built environment and TCE.

## 3. Results

### 3.1. Overall Results

**Figure 1:**
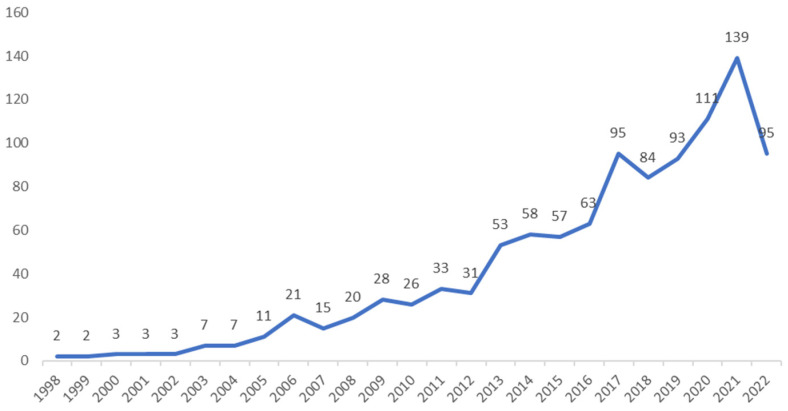
Number of global publications relating to the built environment and TCE (1998–2022).

As shown in Figure 1, from 1998 to 2022, the number of published articles related to TCE in the global built environment showed an increasing trend. Furthermore, this growth was accelerating, indicating that the built environment and TCE have been paid increasing attention. These publications can broadly be divided into three main phases: exploration (1998–2005), development (2006–2016), and continuous acceleration (2017–2022), as described in the next sections.

#### 3.1.1. Exploration Phase (1998–2005)

During the exploration phase, the total number of articles published was 38, and the growth trend of the literature was relatively slow. Among these articles, the largest numbers were published by the United States (11), China (5), and Australia (5). The discussion points of the researchers focused on urban canyons, automobile exhaust, and air quality [39,40,41,42,43]. The discussion of the built environment was relatively minimal. Furthermore, few of these articles are related to CO_2_ emissions from traffic, indicating that the research on the built environment and TCE during this phase was largely exploratory. Although these researchers knew that the built environment may impact CO_2_ emissions, they did not carry out more in-depth research on this topic in particular.

#### 3.1.2. Development Phase (2006–2016)

During the development phase, the annual growth rate of the number of articles was accelerating. A total of 405 articles were published, of which the United States (151), China (73), and the United Kingdom (47) ranked in the top three. From searches of the papers, it was found that the frequency of keywords related to the urban form, built environment, and TCE increased, indicating that the built environment and TCE were paid increasing attention during this phase [44,45,46]. In addition, the Kyoto Protocol came into force in 2005, and there were a series of international conferences and agreements that sought to address the threat posed by climate change. These included the Bali Road Map in 2007, the Copenhagen Accord in 2009, and the Paris Agreement in 2016.

#### 3.1.3. Continuous Acceleration Phase (2017–2022)

The total number of articles published in the acceleration phase was greater than the sum of the previous two phases, indicating that this research has been undergoing a tumultuous period over the past five years, with an average annual volume of more than 90 articles. The country contributing the largest number of these articles, a total of 240, was China, and this was followed by the United States (141) and the United Kingdom (44). During this phase, the research content relating to the built environment and TCE was continuously enriched. Many new methods and technologies continued to emerge and increasing numbers of empirical studies were carried out [47,48,49,50,51,52]. Moreover, the discussion regarding the categories of factors affecting TCE in the built environment became increasingly refined. Because of recent changes in the global environment, this strong development trend should be maintained to provide scientific approaches to sustainable development and environmental protection.

As shown in Table 1, we also analyzed the subject classifications of the three phases. We found that the research focused on environmental sciences (35.66%), environmental studies (23.40%), transportation (21.13%), transportation science technology (18.02%), green sustainable science technology (16.60%), and energy fuels (11.23%). Because each document may belong to more than one category, the total of these proportions is more than 100%. The distribution of discipline categories shows that priority has been given to the problems of the ecological environment, transportation, energy consumption, and sustainable development. In addition, over time, the research on the built environment and TCE has become more interdisciplinary.

### 3.2. Keyword Co-Occurrence Network

**Figure 2:**
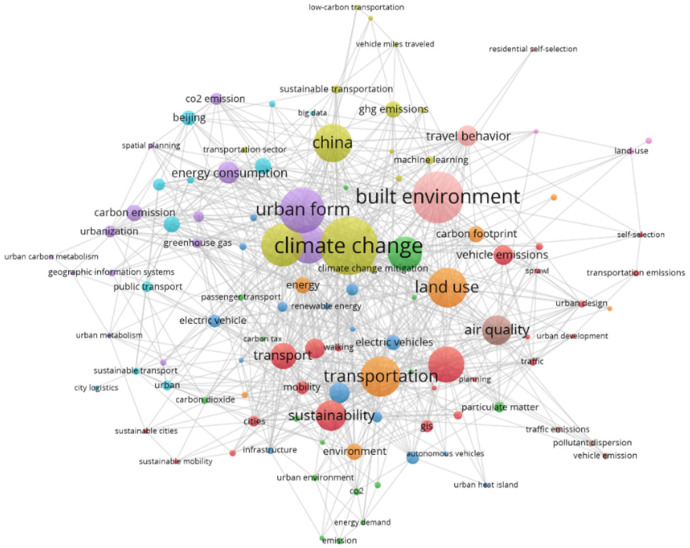
Keyword co-occurrence network.

In this section, we reported the use of VOSviewer and CiteSpace to analyze keywords. Figure 2 shows a co-occurrence map that was generated using VOSviewer with a frequency threshold of 5. Here, the higher the connection intensity of a keyword, the greater its influence. It can be seen from Figure 2 that the five keywords with the highest connection intensity are climate change, urban form, built environment, traffic, and land use. This indicates that these five keywords are closely related to TCE and are worth exploring further.

**Figure 3:**
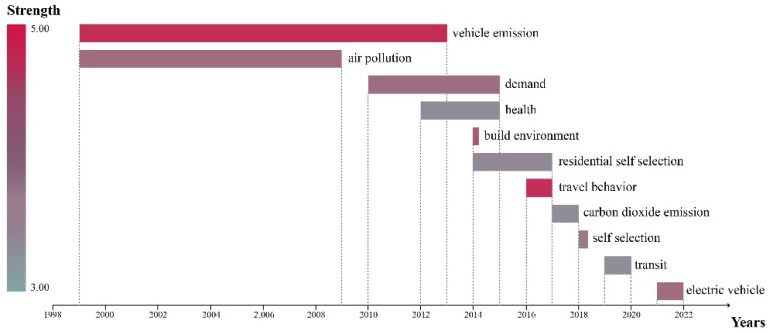
Top-11 keywords with the strongest citation bursts from WOS. The data for this figure were obtained from CiteSpace.

Figure 3 shows the top-11 keywords with the strongest citation bursts from WOS as generated by CiteSpace. On the whole, their emergence shows a step-by-step development. Over time, new hotspots emerge in TCE research, and “vehicle emission”, “travel behavior”, and “built environment” have relatively high emergence values. The emergence values of these three keywords are greater than 4, indicating that they have attracted much attention [53,54,55]. In addition, it can be seen from Figure 3 that “vehicle emissions” and “air pollution” last for a long time, especially the former, which began in 1999 and ended in 2013and had lasted for 14 years. It can thus be extracted that motor-vehicle emissions have been one of the leading research directions in TCE, and many studies have been expanded and extended based on motor-vehicle emissions [56]. In addition, the most recent emergence is “electric vehicle” because electric vehicles may have a significant impact in terms of reducing motor-vehicle carbon emissions. This is a topic worthy of in-depth discussion in the future [57].

### 3.3. Document Co-Citation Network

**Figure 4:**
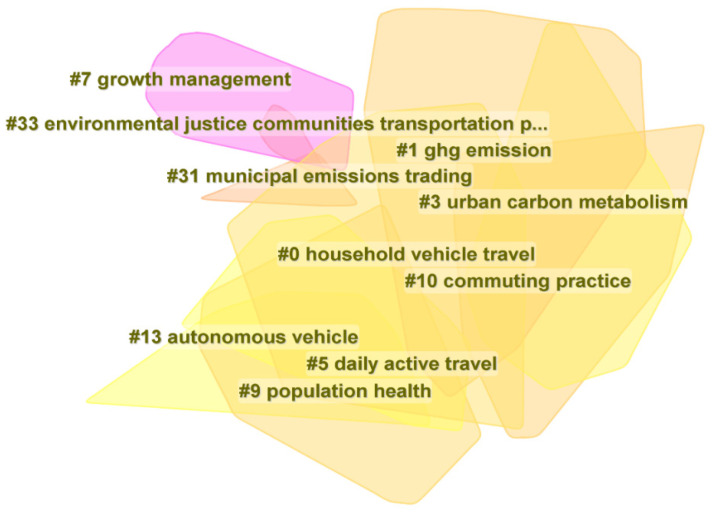
Document co-citation convex hull of clusters. The “#” and number indicate the serial num.

Figure 4 shows the document co-citation network, which consists of 871 cited references and 3435 co-citation links between 1998 and 2022. The silhouette scores of the ten most crucial clusters are all above 0.7, which means that the results are credible (Table 2). The largest cluster—#0 Household vehicle travel and #1 GHG emission—contain 123 and 97 member references, respectively, and are thus slightly more extensive than the others. In these two clusters, the correlation between various factors and carbon emissions is widely discussed [58,59,60]. In addition, the latest cluster is the #13 Autonomous vehicle, which has the characteristics of safety, portability, and low carbon. Some studies show that the combination of autonomous vehicle and infrastructure information can reduce urban emissions by 3.44–19.6% [61,62]. Therefore, automatic driving technology is of great significance for the future development of low carbon cities. In conclusion, with changes in the global environment, TCE and its impact mechanism have become the main focus in the research field of urban carbon emissions.

Based on further analysis of the highly cited literature, Table 3 and Table 4 are drawn from the two perspectives of cited frequency and citation center, respectively. These tables show that Ewing R., Cervero R., Brownstone D., Glaeser E., and Frank L. are the first for both the number of citations and centrality. Ewing R, from the University of Utah, and Cervero R, from the University of California, Berkeley, are cited frequently and centrally, indicating that they are highly influential researchers in the field of TCE.

Among them, Cervero R put forward the “3Ds” theory—representing density, diversity, and design—in a paper published in *Transportation Research Part D* in 1997, which researchers in the field have widely recognized [63]. In a paper published in 2010, he verified that population density is the main factor that reduces vehicle mileage [64]. A paper published by Ewing and Cervero in 2001 demonstrated that, in places with convenient transportation, high density, and high mixing, travel distances are shorter, and they prospectively put forward that idea that the problem of urban parking will become a research hotspot in the future [65,66]. Their argument has attracted much attention and has been continuously expanded and improved over the past 20 years [67,68]. Frank, L., Brownstone, D., and Glaeser, E. are also essential researchers in the field. They have explored and explained the impacts of land-use structure, population density, housing density, distance from the central business district, and other factors on TCE, receiving significant attention [69,70,71].

### 3.4. Factors Influencing TCE in Different Countries and Regions

Research on the factors driving transportation energy consumption and carbon emissions has been carried out in different countries and regions. Many studies have compared different countries or different cities. Croci et al. explored cities in nine different countries. They found that people can travel through high-density cities using non-motorized transportation (e.g., New York City) and decentralized public transportation networks (e.g., New York, London, Mexico City, and Milan), while lower-density cities show heavy use of private cars and higher emissions [77]. Wang et al. compared Xi’an, China, and Bangalore, India. They found that better road conditions, longer commuting distances, and weak public transport services in the outer regions resulted in more car use and high traffic CO_2_ emissions in both cities [78]. According to a study of London and New York by Focas, the suburbs generate the majority of car-based carbon emissions [79]. In addition, many studies have been conducted examining a single country at the provincial or urban level. Yang et al. studied the factors influencing TCE in 33 provinces in China. They found that increasing gross domestic product is the main driving force for the growth of TCE. Furthermore, urban road density and per-capita highway mileage are two other significant factors driving TCE growth, and urban population density has a direct negative impact on TCE [80]. Song et al. studied three cities in the United Kingdom (Cardiff, Kenilworth, and Southampton) and found that age, physical health, and vehicle ownership were significantly related to active transportation [75]. Kang and Oh analyzed the factors influencing TCE in Seoul, South Korea; they found that the number of intersections per kilometer is the most significant factor influencing TCE on the road network [81].

In summary, the factors influencing TCE have been discussed in different countries, and different conclusions have been reached due to different research indicators and dimensions. This is mainly because TCE is not affected by a single factor but is the result of a combination of factors. The current research can only reasonably explain some of these factors. Therefore, further research on the factors influencing TCE is still required.

### 3.5. Methods for Exploring the Built Environment and TCE

In terms of methodology, the two main approaches to exploring this topic are physical spatial and econometric methods. The former mainly examines the spatial differences between different regions using geospatial data; the latter mainly explores the correlations between different factors and dependent variables through population, economic, and other non-spatial data. However, the current trend is to combine the two approaches to reach more comprehensive conclusions [82,83].

In terms of frequency of use, in the previous literature, discrete selection models, regression models, and structural equation models (SEMs) have been widely applied to study the relationship between land use, the built environment, and TCE [84,85,86]. Among these, SEMs are the most widely used [87,88]. SEMs represent a powerful statistical modeling technique that can be used to study the complex interactions between multiple factors; they allow researchers to decompose the total impact of one variable on another variable into direct and indirect effects through other intermediate variables. Compared with simple regression models, the main advantages of SEMs include: (1) modeling intermediate variables so that the total effect is decomposed into direct and indirect effects; (2) considering the measurement errors of all observed variables to ensure accuracy; and (3) identifying causality rather than simple regression coefficients [89].

Zhu et al. constructed an SEM based on natural driving data from 660 private cars in Beijing. They found that the workplace’s impact on fuel consumption is more important than the built environment in which they live, especially the distance from the workplace to the city center [87]. However, it has to be said that, although an SEM can consider intermediary effects when investigating influencing factors, it has limitations in identifying spatial effects; therefore, increasing numbers of researchers are using multi-level Bayesian models [44,67,90]. These methods generally include spatial random effects and conditional autocorrelation models in a framework of hierarchical models. This means that the models can enable a more comprehensive analysis of built-environment factors and spatial effects at different geographical scales. By combining a multi-level Bayesian model with a conditional autocorrelation model, Wang et al. found spatial autocorrelation in the interaction between the built environment and car dependence in Changchun, China [67]. In addition, other researchers have used different methods to explore the built environment and TCE. Wu et al. analyzed the influence of the characteristics of the surrounding built environment on the threshold of travel-related CO_2_ emissions using a gradient-enhancement decision tree. They found that the distance to the nearest bus stop, work density, and land-use diversity were the first three factors affecting CO_2_ emissions [52].

In addition to the above methods, with the progress of research and technology, machine-learning methods have gradually entered the field of vision of researchers [91,92,93]. In recent years, random-forest methods have been used increasingly frequently. Using a random-forest method, He et al. investigated the influence of key variables on the three travel modes of short-distance bus users. They discussed the nonlinear correlations and interactions between different variables [93].

In summary, the research methods used to explore the interactions between the built environment and TCE are constantly developing and improving. Therefore, follow-up research should choose a suitable model as needed and continue to innovate, producing more comprehensive and systematic methods to integrate the advantages of different models and reach more scientific conclusions.

### 3.6. Impact of the Built Environment on TCE

There are various influencing factors affecting urban TCE. This section mainly discusses the built environment’s impact on TCE. We divide the impact of the built environment on TCE into two perspectives: the impacts of functional elements and the impacts of morphological elements; the former represents macro effects, and the latter represent micro effects.

#### 3.6.1. Functional Elements

Land use is fundamentally correlated with TCE; there are differences in the carbon emissions of different land-use types. Once a particular land-use pattern is formed, the corresponding traffic flow will adapt to the land-use pattern and gradually become stable. Therefore, land use determines the carbon emissions of transportation at the macroscopic and functional organization level. Land use mainly affects TCE by changing the travel distance and modes of transport of residents [24,66,73]. The factors of distance and mode include urban size, land-use intensity, and land-use mix.

Urban scale refers to the degree of expansion of the urban area: with the development of urbanization, cultivated land, forests, and grasslands are continuously transformed into construction land. Many studies have shown that maintaining green spaces will help increase CO_2_ carbon sinks, and the development of constructed land will increase CO_2_ emissions [94,95]. In addition, the lengths of roads will also increase with the size of the urban area, which bring the possibility of greater mobility and increase of TCE [96]. However, it must be noted that the relationship between city size and travel behavior is not a simple correspondence but a composite relationship involving multiple factors (e.g., population and economic factors) [97].

Land-use intensity refers to the intensity of land use per unit area. This includes many aspects, such as land-use density, building height, and volume ratio, and it is one of the main characteristics of compact cities [72]. First, high-intensity development is the basis of the construction of public transport facilities, and this improves the possibility of centralized provision of public transport services for urban areas [98]. Second, the compact urban landscape can reduce the transport needs of residents [99,100]. Megill Legendre, found that, in metropolitan areas, increased compactness may reduce family vehicle mileage by 5–12%, or even as much as 25% [101].

**Figure 5:**
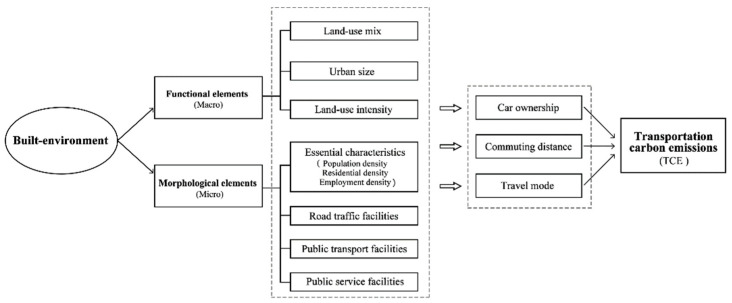
The impact of built-environment on transportation carbon emissions.

Land-use intensity and land-use mix often jointly affect TCE. It is generally believed that high density and highly mixed land use can shorten travel distances, thus increasing the possibility that people use public transport, walking, and cycling [102]. In addition, some studies have found that areas with highly mixed land use have lower car ownership [67]. However, some researchers have put forward a different point of view, believing that, with increasingly mixed land use, the possibility of owning a car will increase. This is because people living in compact mixed land-use areas have to travel shorter distances than those living in low-compact land-use areas, and this reduces their costs for travel and car ownership, therefore increasing TCE [68]. The impact mechanism of built environment on TCE is shown in Figure 5.

#### 3.6.2. Morphological Elements

Previous studies have mainly explored the indirect effects of the morphological characteristics of the built environment on urban TCE at the micro level. The impact of morphological elements includes four main aspects: essential characteristics, road traffic facilities, public transport facilities, and public service facilities. Among them, essential characteristics includes residential density, employment density, and population density. It is generally believed that residential density is negatively correlated with TCE [26,103,104]. That’ because communities with high residential density may have better living facilities, people’s needs can be met in a shorter distance, then reducing the need for motorized transport. Using a Bayesian multi-level analysis framework based on conditional autoregressive estimation and spatial random effects, Hong and Qing found that a 1% increase in residential density can reduce TCE by 0.16–0.37% [26]. Most reports conclude that there is a negative correlation between employment density and TCE [44,86,87]. Wang et al. found that an increase in employment density helps to reduce the average speed of vehicles, increase fuel efficiency, and reduce transportation energy consumption [105]. Nevertheless, some reports have raised questions about this opinion [90,106].

Some studies have found that, although employment density can help reduce TCE to a certain extent, increasing employment density, especially in residential areas, may increase TCE [90]. The reason is that the high housing prices and rents brought about by an increase in employment density make it impossible for many residents to live in these communities, thus leading to longer commuting distances. Population density has always been a research hotspot regarding factors influencing TCE [46,55,83]. The conclusions of studies on the impact of population density on TCE also have two tendencies.

On the one hand, some studies have found that lower population density leads to longer travel distances for the reasons are similar to residential density [107]. Communities with higher population density are equipped with more convenient service facilities and transportation systems, which reduces the need for high-energy travel. Using multi-level SEM analysis, Lee and Lee found that a 10% increase in population-weighted density will lead to a 4.8% reduction in household TCE [46]. Seyed also found that a 10% increase in population density was correlated with 3.5%, 1.5%, and 1.4% reductions in transport carbon emissions in Montreal, Quebec, and Sherbrooke [83].

On the other hand, some reports have suggested that increasing population density may promote TCE for the reason that population density mainly indirectly affects traffic emissions through car ownership and commuting distance [108,109]. It means that if only considering the increasing population density, but ignoring the variation of commuting distance or car ownership, as a matter of fact, it may not effectively reduce TCE: it may even promote it [55]. In addition, many studies have shown that residents living in denser urban environments are more inclined to take more long-distance leisure trips to relieve the pressure caused by compact living environments, and this generates more TCE [110].

Previous discussions on road transport facilities have focused on road networks and intersections [79,82,84,111]. Regarding the road network, this mainly affects TCE through the road length and road network density. With the continuous expansion of an urban area and the continuous improvement of road infrastructure, the total urban road mileage will show a growing trend. If the density of the road network increases, this can also provide residents with more choices of travel, shorter travel distances, and greater alleviating of congestion. Sun et al. found that a 1% increase in road length in a residential area will reduce the likelihood of choosing a private car by 0.23%. Similarly, a 1% increase in road length in an employment area will reduce the average commuting distance by 0.49% by bus and increase the commuting distance by 0.72% by bicycle [106]. Nevertheless, with an increasing number and density of roads, the carrying capacity of road vehicles will also increase, and the degree of suburbanization will become higher, which will lead to more TCE. A study by Xie et al. of 283 prefecture-level cities in China showed that an increase in road length positively impacts the urban CO_2_ emissions of large and medium-sized cities [112]. In addition, intersection density also has an important impact on TCE. Many studies have found that people in residential areas with high intersection density are less likely to own cars or travel by motor vehicle [68,106]. On the one hand, high-density intersections are more pedestrian friendly; on the other hand, these areas are generally hubs or centers of public transport, which will encourage residents to travel by public transport. Therefore, areas with high intersection density are conducive to reducing TCE.

The impact of public transport facilities on TCE mainly depends on the number, density, and accessibility of public transport stations. Most research results show that public transport facilities are conducive to reducing TCE [67,82,102,113,114]. A dense distribution of public transport will increase the accessibility of bus stops to residents, have a substitution effect on cars, reduce the travel in and congestion from private cars, and reduce car ownership, thereby reducing energy consumption per capita. Hu et al. found that, if 15% of private motor trips was replaced by public transport, the total transport energy consumption would be reduced by at least 60% [115]. Zhao et al. also found that the length of subway lines is negatively correlated with personal transportation energy consumption. Every 1% increase in subway-line length reduces personal transportation energy consumption by 1.61% [107]. Transit-oriented development has been carried out in many large cities so that residents can meet most of their living needs near bus stops. This reduces their need to travel longer distances, which is conducive to reducing TCE [113,116,117].

Public service facilities mainly refer to services needed in residents’ daily lives, such as education, medical care, transportation, leisure, sports, and financial services. Most studies conclude that the higher the density of public service facilities, the higher their accessibility to the community [68,118,119]. Therefore, reducing the TCE caused by residents meeting their living needs is helpful. Studies have found that a dense distribution of schools and supermarkets has a more critical impact on residents’ TCE than hospitals and banks because the former are closely related to regular travel while the latter are only occasionally used [118]. However, Ma et al. found that the configuration of public service facilities may increase the non-working travel distance [120]. The difference in these conclusions may be related to the different types and levels of public service facilities. In addition, some articles discuss the impact of the distribution of parking facilities on TCE. Christiansen explored the impact of parking availability in the workplace on commuting mode choices. He found that limited parking facilities in the workplace can reduce the use of cars [121]. Some researchers have also paid attention to the impact of parking facilities in residential areas. Weinberger (2012) used a generalized linear model to predict the proportion of residents driving to work [122]. Considering the distribution of parking facilities in communities, Guo (2013) used Google Street View to measure the parking supply of 770 households in New York City. They both found that family parking restrictions help reduce car ownership and use [123].

## 4. Discussion

As the problem of urban TCE has received widespread attention, many researchers have explored the impacts of different factors on TCE. However, the current research still lacks a systematic analytical framework, and its scale needs to be increased. Moreover, there is an urgent need for new technologies and methods to support more scientific and practical empirical research to promote the reduction of carbon emissions in different countries and cities worldwide. Some suggestions from this review are given as follows.

Foremost, it is necessary to establish a comprehensive research system to quantify the degree of coordination and conflict. With the acceleration of global urbanization, researchers in different regions and countries have conducted many studies on the factors affecting TCE [78,97]. Due to the complexity and relevance of the influencing factors, the differences in models, the indicators used, and the conclusions of the research on the influencing factors in different regions are not always consistent, and there may be conflicts [90,109]. For example, whether self-selection effects or socioeconomic attributes are considered will significantly impact the results. Therefore, it is necessary to establish a complete research system to quantify the degree of coordination and conflict. It will improve the applicability of built environment indicators in different scenarios and draw conclusions that are reached more reasonable and scientific.

In addition, it is important to increase the scale of research. The examination of the existing literature records found that the research on the relationship between the built environment and TCE has mainly focused on a single city or many cities in a single country [75,79,80], but few have compared the perspectives of large and small cities or the cities in developing or developed countries. Meanwhile, the studies have also mainly discussed TCE from the perspective of urban space. Therefore, subsequent research could be carried out on the correlations between various travel purposes and the built environment. Then, the scale of the research should be increased to better consider the correlations between the built environment and TCE.

Moreover, optimization of methods and techniques can benefit research a lot. From a theoretical point of view, the relationship between the built environment and TCE is mainly regulated by individual travel behavior. Therefore, a large number of studies have measured the relationship between the built environment and personal travel behavior using a variety of methods and techniques, including travel mode choice, travel schedule, time, and destination [87,90,93]. However, because of the complexity of residents’ travel patterns, existing technologies and methods cannot fully guarantee the consistency of the processing of personal travel behavior. Therefore, techniques involving big data, machine learning, and other new technologies should be integrated more deeply into this field of research to establish more refined models and methods to promote its development.

Finally, future research should explore new possibilities based on the interaction between urban architectural environment and TCE. For example, more and more authors have changed their research perspective from linear regression of traditional building environment to nonlinear relationship [22]. Meanwhile, future research should focus on the complexity and diversity of emission impacts, and consider fully the impact mechanism of influencing factors such as residents’ travel attitude [124], air quality [125] and public health awareness [126], to promote the development of future low-carbon cities, meet the needs of residents, and improve environmental quality.

## 5. Conclusions

Through bibliometrics, in this study, we analyzed the literatures to produce a knowledge map of the correlations between the urban built environment and TCE. We summarized the published trends, keywords, and the characteristics of literature co-citation. In addition, we also combed through the factors influencing TCE in different countries and the methods used in analyzing TCE. Past research results and future development trends in this field have been summarized. Our conclusions can be presented as follows.

The research on the correlation between the built environment and TCE shows that the total amount of literature has been experiencing a continuous growth trend. This growth can be divided into three development phases: exploration, development, and continuous acceleration. In addition, TCE, as a global problem, has been widely considered in different regions and countries worldwide. The factors that affect TCE vary from country to country.

The visual analysis of keywords shows that climate change, urban form, built environment, transportation, and land use are topics of intensive research. From the keyword burst map, it was found that the emergence of these keywords from 1998 to 2022 showed a step-by-step development. New research hotspots continue to emerge, among which vehicle emissions is the most investigated topic.

The built environment’s impact on TCE comes from many factors and is a complex process. First, from a macro point of view, the functional elements of the built environment, including city size, land-use intensity, and land-use mix, have an important impact on TCE. Second, from a micro point of view, the impact of the morphological elements of the built environment on TCE is closely related to the spatial distributions of urban density, road traffic, public transport, and public services. The impact of the built environment on the traffic environment is mainly caused by the indirect impacts of these factors on residents’ travel distance and behavior, as well as the number of private cars.

## Figures and Tables

**Table 1:** Subject classifications from WOS and their frequencies.

Subject Classification	Frequency	Proportion
Environmental Sciences	378	35.66%
Environmental Studies	248	23.40%
Transportation	224	21.13%
Transportation Science Technology	191	18.02%
Green Sustainable Science Technology	176	16.60%
Energy Fuels	119	11.23%
Engineering Civil	114	10.76%
Engineering Environmental	106	10.00%
Economics	96	9.06%
Meteorology Atmospheric Sciences	81	7.64%
Urban Studies	81	7.64%
Regional Urban Planning Health	64	6.04%
Geography	53	5.00%
Construction Building Technology	45	4.25%
Public Environmental Occupational Health	35	3.30%

**Table 2:** Summary of the largest ten clusters.

Cluster ID	Size	Silhouette Score	Label	Mean (Cite Year)
0	123	0.782	Household vehicle travel	2012
1	97	0.817	GHG emission	2013
3	52	0.850	Urban carbon metabolism	2018
5	32	0.958	Daily active travel	2019
6	28	0.922	Direct eddy-covariance measurement	2012
7	27	0.985	Growth management	1998
9	22	0.938	Population health	2016
10	20	0.965	Commuting practice	2014
13	15	0.985	Autonomous vehicle	2020
31	6	0.987	Municipal emissions trading	2011
33	5	0.996	Environmental justice communities Transportation planning	2009

**Table 3:** Top-10 co-citation frequencies of the papers.

Citations	Reference	Cluster ID	Primary Outcomes
115	Ewing R. (2010) [66]	0	DA(−); LM(−); ID(−)
63	Cervero R. (1997) [63]	0	RD(−); LM(−); PD(−)
58	Brownstone D. (2009) [69]	0	RD(−)
53	Ewing R. (2001) [65]	0	AA(−); RD(−); LM(−)
40	Glaeser E. (2010) [71]	1	LP(−)
37	Cervero R. (2010) [64]	0	PD(−)
36	Newman P. (1989) [72]	1	LM(−)
35	Handy S. (2005) [73]	0	RD(−); LM(−)
26	Frank L. (2000) [42]	0	RD(−); ED(−); LM(−)
26	Ewing R. (2008) [74]	1	CR(−)

Notes: (1) Capital letters represent abbreviations for different variables: RD = residential density, PD = pedestrian-oriented designs, AA = area accessibility, LM = land use mix, DA = destination accessibility, LP = land policy, PD = population density, ED = employment density, ID = intersection density, CR = compactness ratio. (2) “−” means negative correlation with TCE.

**Table 4:** Top-10 co-citation centrality of the papers.

Centrality	Reference	Cluster ID	Primary Outcomes
0.09	Ewing R. (2001) [65]	0	AA(−); RD(−); LM(−)
0.08	Ewing R. (2010) [66]	0	DA(−); LM(−); ID(−)
0.06	Frank L. (1994) [70]	7	RD(−); LM(−)
0.05	Cervero R. (2010) [64]	0	PD(−)
0.05	Cervero R. (1997) [63]	0	RD(−); LM(−); PD(−)
0.04	Handy S. (2005) [73]	0	RD(−); LM(−)
0.04	Frank L. (2000) [42]	0	RD(−); ED(−); LM(−)
0.03	Brownstone D. (2009) [69]	0	RD(−)
0.03	Brand C. (2013) [75]	2	WD(+); PO(+)
0.03	Bagley M. (2002) [76]	0	PO(+); PA(+); DC(+)

Notes: (1) Capital letters represent abbreviations for different variables: RD = residential density, PD = pedestrian-oriented designs, AA = area accessibility, LM = land use mix, DA = destination accessibility, PD = population density, ED = employment density, ID = intersection density, WD = working distance, PO = private car ownership, PA= parking lot availability, DC = distance to CBD. (2) ”+” means positive correlation with TCE, “−” means negative correlation with TCE.

## Data Availability

Data are contained within the article.
